# Rational Design of Modified Oxobacteriochlorins as Potential Photodynamic Therapy Photosensitizers

**DOI:** 10.3390/ijms20082002

**Published:** 2019-04-24

**Authors:** Marta Erminia Alberto, Bruna Clara De Simone, Emilia Sicilia, Marirosa Toscano, Nino Russo

**Affiliations:** Dipartimento di Chimica e Tecnologie Chimiche, Università della Calabria, 87036 Rende, Italy; malberto@unical.it (M.E.A.); emilia.sicilia@unical.it (E.S.); m.toscano@unical.it (M.T.)

**Keywords:** oxobacteriochlorins, PDT, TDDFT, spin-orbit coupling, heavy atom effect

## Abstract

The modulation of the photophysical properties of a series of recently synthetized oxobacteriochlorins with the introduction of heavy atoms in the macrocycles, was investigated at density functional level of theory and by means of the time-dependent TDDFT formulation. Absorption frequencies, singlet-triplet energy gaps and spin-orbit coupling (SOC) constants values were computed for all the investigated compounds. Results show how the sulfur- selenium- and iodine-substituted compounds possess improved properties that make them suitable for application in photodynamic therapy (PDT).

## 1. Introduction

Photodynamic therapy (PDT) is used in cancer treatment due to its mini-invasive nature, limited side effects, easy procedure and short treatment time in respect to surgery or chemotherapy [1,2,3,4,5]. PDT is based upon the so-called photodynamic effect, that can be defined as the tissue destruction or damage by the combined action of a photosensitizer, a visible or near visible light, and molecular oxygen. The key cytotoxic agent is the highly reactive singlet exited oxygen (O_2_, ^1^Δ_g_) produced in a photophysical cycle (type II photoexcitation process) which can be briefly described as it follows [6]: Absorption of light with the appropriate wavelength by a photosensitizer in its ground state (S_0_) leads to the excitation of one electron into a higher-energy orbital. The excited singlet (S_1_) that is formed may undergo an ‘intersystem crossing’ (ISC) to generate a more stable excited triplet state (T_1_). The long lifetime of the triplet state allows to transfer its energy to the molecular oxygen (O_2_), which passes from its triplet ground state (^3^Σ_g_) to the singlet excited state (^1^Δ_g_). The efficiency of the ISC process strictly depends on the amplitude of the spin-orbit coupling singlet constants (SOC) [7] and it is possible only if the lowest triplet excited state (T_1_) lies above the energy needed to produce the molecular singlet oxygen (0.98 eV). In order to better penetrate into the tissue, the photosensitizer absorption wavelength should fall in the so called PDT absorption window that is between 500 to 800 nm. To improve the penetration of the light to the tissue, many scientists work on excitation with longer wavelength at near-IR or even longer wavenumbers [5]. In this spectroscopic region, even if the light deeply penetrates into the tissues it does not produce side effects due to the high frequency vibrations. In addition, the photosensitizer must be non-toxic in the dark, not subjected to intermolecular aggregation phenomena (which would diminish its phototherapeutic efficacy), and soluble in aqueous environment.

Although research in this area has increased significantly in recent years [4,5,6], the number of molecules, that have passed clinical trials to be used in medical protocols, are few and essentially based on porphyrin-like structures. Among these, we mention Foscan^®^ (Temoporfin) [8], Photofrin^®^ (porfimer sodium) [9], and Laserphyrin^®^ (Talaporfin) [10]. Second generation photosensitizers such as Lutrin^®^ (Lutetium texaphyrin) are in different clinical phases [11]. The success of the porphyrin-like systems as PDT agents is due to the fact that, as it is required, they are not toxic, easily soluble in physiological solutions and able to absorb into the spectral region of the therapeutic window. Furthermore, their long-wavelength absorption Q band can be tuned easily introducing different chemical groups at distinct sites around the perimeter of the hydroporphyrin skeleton. In particular, the presence of auxochromes positioned along the bisection axis of pyrrole rings causes batochromic shifts with respect to the unsubstituted chorines or bacteriochlorins.

Recently, a series of oxo- and thioxo- substituted bacteriochlorins [12,13] have been synthetized and their photophysical properties determined. Moreover, other photosensitizers with different chemical structures, squaraine [14], BODIPY [15], aza-BODIPY [16] and BOIMPY [17] have been proposed for their use in PDT on the basis of cytotoxicity tests. Some of these works underline the possibility to redshift the absorption Q band as a function of the substituents position in the macrocyclic skeleton, being this, one of the most important requirements to satisfy in designing new PDT drugs.

In this scenario, modern methods of theoretical and computational chemistry can offer significant aids for a reliable and rational design of targeted drugs by shortening the usually long chain from chemical synthesis to clinical approval. In particular, density functional based methods (DFT) are able to predict the structural, energetic and spectroscopic properties of a great number of complex chemical systems conjugating reasonable computational effort with chemical accuracy.

In this work we will show how, by using DFT, it is possible not only to correctly reproduce a series of photophysical properties, but also predict their change as a result of a targeted substitution of atoms or functional groups. Starting from some recently synthesized oxobacteriochlorins [12], the calculation of their absorption spectra, singlet-triplet energy gaps and spin-orbit couplings constants allowed to suggest chemical modifications that fulfill the criteria necessary to propose them as photosensitizers in photodynamic therapy.

## 2. Results and Discussion

**Scheme 1:**
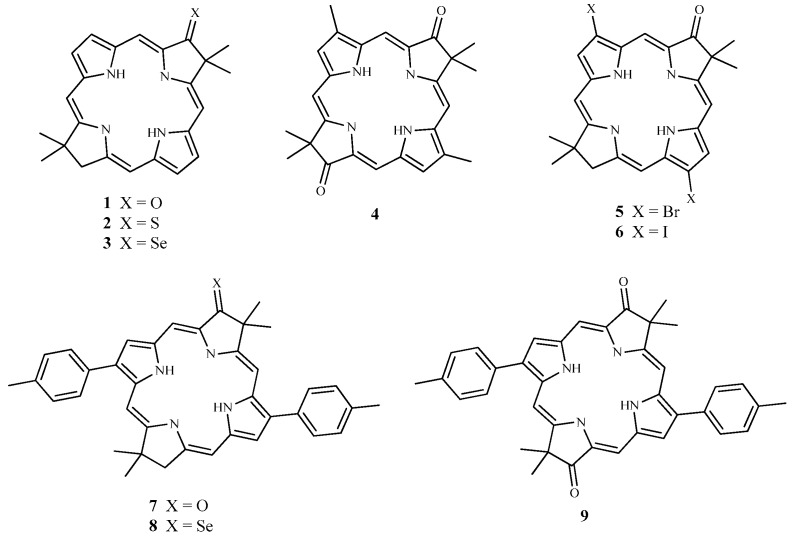
Schematic representation of compounds investigated in this work.

The ground and excited singlet and triplet states optimized structures (see Table S1 for Cartesian coordinates) present a planar conformation of the hydroporphyrin macrocycle in all studied systems (see Scheme 1).

In order to evaluate the effects of the heavy atom on the photophysical properties of the considered systems, vertical excitation energies at TDDFT level of theory have been calculated (see Table 1 and Figure S1). From the obtained values it results a batochromic shift in going from compounds **1**, with an oxo group (599 nm), to **2** (623 nm) and **3** (678 nm) in which S and a Se atoms, respectively substitute the oxygen one.

**Figure 1:**
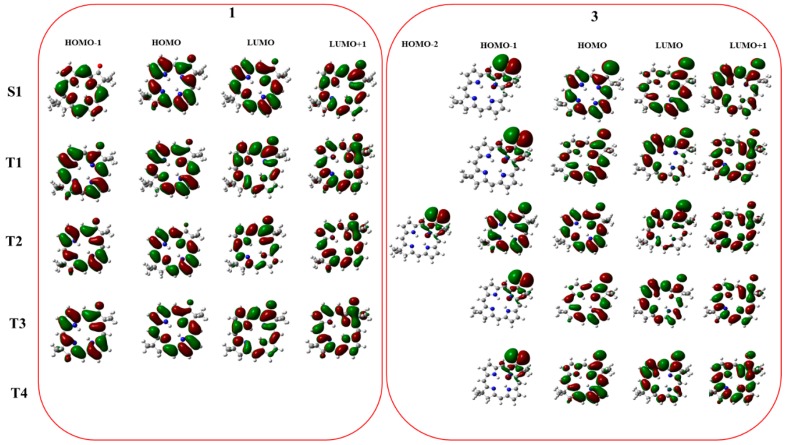
Molecular orbital composition for the excited states involved in the singlet and triplet excitation for molecules for **1** and **3** molecules.

The dioxo molecule (**4**) shows a Q band at 602 nm meaning that the addition of a second oxo group does not change the absorption behavior significantly. The same situation is found in systems **7** and **9**, where two phenyl groups are present in the phorphyrin-like skeleton. In all systems, the transitions involve the HOMO and LUMO orbitals and are π-π in nature. This can be inferred from the molecular orbital pictures of all the three compounds, in which a π delocalization on the entire macrocyclic ring (see Figure 1) is present. Several triplet states have been localized above the low-lying excited singlet one. In particular, three triplet states have been found in unsubstituted molecules (**1**, **4–7**, **9**) while four ones appeared when a sulfur or a selenium atom replaces the oxo group (**2**, **3**, **8**). T_1_ derives from a HOMO-LUMO transition while T_2_ and T_3_ are originated by an electron transfer involving different orbitals (see Table 1). T_4_, present in the thioxi and selenoxi containing molecules, is almost degenerate with the S_1_ state. For all systems, the energy gap between the ground state and the low-lying triplet (T_1_) assumes values higher than that required to activate molecular oxygen from the ^3^Σ_g_ electronic state to ^1^Δ_g_ one (0.98 eV) and therefore satisfies one of the criteria necessary for an efficient PDT photosensitizer (see Table 1).

As previously mentioned, the photosensitizers active in PDT must possess an efficient singlet-triplet intersystem crossing whose kinetic constant can be derived from the Fermi golden rule throughout the following expression [7]:

$$k_{ISC}\left(S_{1}\to T_{j}\right)=\frac{2\pi }{\hbar }\frac{{\langle S_{1}|H_{SO}|T_{j}\rangle }^{2}}{\sqrt{4\pi \lambda k_{B}T}}\underset{n}{\sum }\frac{S_{k}^{n}}{n!}exp\left(-S_{k}\right)exp\left[-\frac{{\left(\Delta E_{S-T_{j}}+n\hbar \omega_{k}+\lambda_{s}\right)}^{2}}{4\lambda k_{B}T}\right]$$

where $\langle S_{1}|H_{SO}|T_{j}\rangle$ are the spin-orbit matrix elements, *ω_k_* the high-frequency vibrational modes and Δ*E_S-T_* is energy gap between the *S*_1_ and *T_j_* states at their equilibrium geometries. In this formulation, the Franck–Condon-weighted density of states is estimated by the Marcus theory for the non-radiative transition from *S*_1_ to each triplet excited state (*T_j_*) lying below.

From the reported equation, it can be inferred how the ISC efficiency depends mainly on the values of the spin orbit couplings other than the singlet-triplet energy gaps for the involved ISC. For all the investigated compounds, we have decided to compute the SOCs concerning the *S*_1_ → *T_j_* transitions. The same procedure has been adopted for the selenium containing molecules **3** and **8** for which the S_2_ state is populated (see Table 1). This should mean that, in agreement with the Kasha rules [18], after the excitation, which populates the *S*_2_ state, this state decays on *S*_1_ through a fast internal conversion process (IC).

The computed $\langle S_{1}|H_{SO}|T_{j}\rangle$, together with the relative Δ*E_S1-Tj_* energy gaps are collected in Table 2.

From that Table it clearly appears that the presence of heavy atoms in the porphyrin-like skeleton greatly influences the amplitude of the spin orbit coupling constants. In fact, in going from system **1** (oxo), to **2** (thioxo), to **3** (selenoxo), the $\langle S_{1}|H_{SO}|T_{1}\rangle$ increases significantly assuming values of 114.7 and 685.8 cm^−1^ for **2** and **3**, respectively. The same behavior is found for compounds **7** (0.2 cm^−1^) and **8** (675.4 cm^−1^). It is interesting to note that for these two last compounds, having a *T*_4_ state with energy similar to that of *S*_1_, the SOCs values for the *S*_1_ → *T*_4_ transitions have quite high values (52.2 and 239.5 cm^−1^ for **2** and **3**, respectively).

The presence of halogen atoms also contributes to increase the $\langle S_{1}|H_{SO}|T_{1}\rangle$ matrix element, especially in the case of iodine (17.4 cm^−1^). While for the other examined systems the values of the other S1–Tj SOCs are negligible, for the iodine-containing system (**6**) the $\langle S_{1}|H_{SO}|T_{2}\rangle$ and $\langle S_{1}|H_{SO}|T_{3}\rangle$ matrix elements increase considerably compared to those of the unsubstituted system (see Table 2).

These trends can be rationalized using the El Sayed rules [19] that establish that SOC values increase significantly if a symmetry change of the molecular orbitals involved in the transitions, occurs. The orbital pictures of singlet (S_1_) and triplets (T_1_–T_4_) for systems **1** and **3** are reported in Figure 1.

For the unsubstituted oxobacteriochlorin (see Table 1) S_1_ and T_1_ transition involves the HOMO-LUMO orbitals that are both π-π * in nature. Although for T_1_ and T_2_ the transitions are due to HOMO-1→LUMO and HOMO→LUMO+1, respectively, Figure 1 shows that all these orbitals are of π type. The presence of selenium in **3** affects significantly the ISC in the S_1_→T_1_ and S_1_→T_4_ transitions since the orbital compositions change becoming nπ→ππ with different percentage (Table 1). In accordance with the El Sayed rules [19], the SOCs values should be high. In other considered intersystem crossings, the transition involves orbitals with almost the same symmetry in agreement with the low SOC values obtained by our calculations.

Same correlations between orbital nature and SOC amplitudes have been previously found for S, Se and Te-substituted deoxyguanosines [20] and thymidines [21]. Furthermore, the heavy atom effects have been explored in the case of halogen substituted aza-BODIPY [22], BOIMPY [23] and metal containing tetraphenylporphyrin [24].

Considering the computed spin-orbit coupling constants, we can suggest the following possible faster deactivation paths for the considered ISC processes:

$S_{0}\overset{A}{\to }S_{2}\overset{\mathrm{IC}}{\to }S_{1}\overset{\mathrm{ISC}}{\to }T_{1}$ or $S_{0}\overset{A}{\to }S_{2}\overset{\mathrm{IC}}{\to }S_{1}\overset{\mathrm{ISC}}{\to }T_{4}\;\overset{\mathrm{IC}}{\to }T_{1}$ for compound **3**;
$S_{0}\overset{A}{\to }S_{1}\overset{\mathrm{ISC}}{\to }T_{1}$ or $S_{0}\overset{A}{\to }S_{1}\overset{\mathrm{ISC}}{\to }T_{4}\;\overset{\mathrm{IC}}{\to }T_{1}$ for compounds **2** and **8**;
$S_{0}\overset{A}{\to }S_{1}\overset{\mathrm{ISC}}{\to }T_{1}$ or $S_{0}\overset{A}{\to }S_{1}\overset{\mathrm{ISC}}{\to }T_{2}\;\overset{\mathrm{IC}}{\to }T_{1}$ for compound **6**;
$S_{0}\overset{A}{\to }S_{1}\overset{\mathrm{ISC}}{\to }T_{1}$ for all other systems.

An accurate prediction of $k_{ISC}\left(S_{1}\to T_{j}\right)$ requires that the adiabatic singlet-triplet energy gaps are taken into consideration as well as the calculation of the Franck–Condon weighted density of states. Work is in progress in this direction.

## 3. Materials and Methods

All the computations have been carried out by using the Gaussian 09 [25] code employing DFT and its time dependent formulation TDDFT [26]. The hybrid B3LYP [27,28] exchange–correlation functional has been used in conjunction with the 6-31+G* basis set for all the atoms and the SDD pseudopotential [29] for iodine. No symmetry constraints have been imposed during the geometry optimizations.

Computations have been performed in toluene to mimic the experimental environment, by using a dielectric constant of ε = 2.37. The integral equation formalism for the polarizable continuum model (IEFPCM) [30], which corresponds to a linear response in nonequilibrium solvation, has been employed. Spin–orbit matrix elements $\langle S_{1}|H_{SO}|T_{j}\rangle$ have been computed with the DALTON code [31] by using the spin–orbit coupling operators for effective core potentials with an effective nuclear charge [32] for iodine atoms containing systems. The atomic mean field approximation [33] has been used in the other cases. For this purpose, the B3LYP functional has been employed in conjunction with the cc-pVDZ basis set for all the atoms except for iodine for which the corresponding pseudopotential has been adopted.

The choice of the computational protocol has been suggested by our previous benchmarks in computing different photophysical properties for several compounds [34,35,36,37,38,39].

## 4. Conclusions

Stimulated by the recent synthesis of a series of oxo- and dioxobacteriochlorine that should produce singlet oxygen under UV-Vis irradiation, we have computed the main photophysical properties of such compounds relevant in photodynamic therapy. In addition, we have demonstrated that the inclusion of a heavy atom (S, Se, I) in the skeleton of these systems strongly improves the spin-orbit coupling constant values, and consequently the efficiency of the intersystem crossing, ensuring high yield of the cytotoxic singlet molecular oxygen agent.

All the studied systems show absorption wavelengths that fall in the so-called photodynamic window and possess a singlet-triplet energy gap higher than that necessary to generate ^1^Δ_g_ O_2_ (0.98 eV).

The computed SOCs have values higher than that of Foscan^®^ already used as photosensitizer in the PDT clinical protocols.

We hope that our work can stimulate the synthesis and the characterization of new possible PDT drugs.

## Figures and Tables

**Table 1:** B3LYP/6-31+G* vertical excitation energies, ΔE in eV and (nm), oscillator strengths *f* and main orbital transitions for the studied compounds (in toluene). H = HOMO; L = LUMO. Experimental values are taken from ref. [12].

Molecule	State	ΔE		*f*	Transitions	λ_exp_
**1**	S_1_	2.07	(599)	0.247	H→L, 87%	690
	T_1_	1.31	(948)		H→L, 93%	
	T_2_	1.36	(910)		H−1→L, 84%	
	T_3_	2.01	(617)		H→L+1, 87%	
**2**	S_1_	1.99	(623)	0.244	H→L, 80%	
	T_1_	1.22	(1013)		H→L, 81%	
	T_2_	1.38	(898)		H-1→L, 59%	
	T_3_	1.67	(741)		H→L+1, 75%	
	T_4_	1.82	(682)		H-2→L, 57%	
**3**	S_1_	1.83	(678)	0.000	H-1→L, 77%	
	S_2_	1.95	(636)	0.279	H→L, 79%	
	T_1_	1.15	(1076)		H→L, 85%	
	T_2_	1.38	(898)		H-2→L, 55%	
	T_3_	1.54	(806)		H-1→L, 70%	
	T_4_	1.62	(764)		H→L+1, 70%	
**4**	S_1_	206	(602)	0.200	H→L, 82%	680
	T_1_	1.29	(963)		H→L, 66%	
	T_2_	1.44	(863)		H-1→L, 58%	
	T_3_	1.93	(641)		H→L+1, 84%	
**5**	S_1_	2.02	(613)	0.333	H→L, 89%	703
	T_1_	1.29	(964)		H→L, 98%	
	T_2_	1.33	(929)		H-1→L, 87%	
	T_3_	2.00	(620)		H→L+1, 87%	
**6**	S_1_	2.02	(614)	0.362	H→L, 89%	
	T_1_	1.29	(964)		H→L, 100%	
	T_2_	1.33	(933)		H-1→L, 90%	
	T_3_	2.00	(619)		H→L+1, 87%	
**7**	S_1_	1.99	(624)	0.457	H→L, 86%	709
	T_1_	1.26	(988)		H→L, 88%	
	T_2_	1.38	(896)		H-1→L, 80%	
	T_3_	1.98	(627)		H→L+1, 86%	
**8**	S_1_	1.83	(679)	0.000	H-2→L, 60%	
	S_2_	1.89	(655)	0.373	H→L, 82%	
	T_1_	1.12	(1109)		H→L, 87%	
	T_2_	1.40	(883)		H-1→L, 47%	
	T_3_	1.54	(806)		H-2→L, 53%	
	T_4_	1.59	(781)		H→L+1, 72%	
**9**	S_1_	2.01	(616)	0.355	H→L, 84%	695
	T_1_	1.23	(1007)		H→L, 66%	
	T_2_	1.45	(858)		H-1→L, 63%	
	T_3_	1.95	(635)		H→L+1, 85%	

**Table 2:** Spin–orbit matrix elements (cm^−1^) and singlet-triplet energy gaps (eV) calculated at the B3LYP/6-31G(d) level of theory.

Cmpd	$\left|\langle \Psi_{S_{1}}|{\hat{H}}_{so}|\Psi_{T_{1}}\rangle \right|$	ΔE_S1-T1_	$\left|\langle \Psi_{S_{1}}|{\hat{H}}_{so}|\Psi_{T_{2}}\rangle \right|$	ΔE_S1-T2_	$\left|\langle \Psi_{S_{1}}|{\hat{H}}_{so}|\Psi_{T_{3}}\rangle \right|$	ΔE_S1-T3_	$\left|\langle \Psi_{S_{1}}|{\hat{H}}_{so}|\Psi_{T_{4}}\rangle \right|$	ΔE_S1-T4_
**1**	2.6	0.8	1.9	0.7	0.4	0.1		
**2**	114.7	0.8	2.2	0.6	0.6	0.3	52.2	0.2
**3**	685.8	0.7	8.4	0.5	1.5	0.3	239.5	0.2
**4**	2.1	0.8	3.2	0.6	1.0	0.1		
**5**	2.8	0.73	2.1	0.69	0.9	0.0		
**6**	17.4	0.7	71.1	0.69	35.2	0.0		
**7**	0.2	0.8	0.2	0.61	0.1	0.0		
**8**	675.4	0.7	9.2	0.43	3.9	0.3	258.6	0.2
**9**	0.1	0.8	0.1	0.56	0.2	0.1		

## Supplementary Materials

Supplementary materials can be found at https://www.mdpi.com/1422-0067/20/8/2002/s1.

## References

[1] Agostinis P., Berg K., Cengel K.A., Foster T.H., Girotti A.W., Gollnick S.O., Hahn S.M., Hamblin M.R., Juze-niene A., Kessel D. (2011). Photodynamic Therapy of Cancer: An Update. CA Cancer J. Clin..

[2] Dolmans D.E., Fukumura D., Jain R.K. (2003). Photodynamic therapy for cancer. Nat. Rev. Cancer.

[3] Allison R.R., Bagnato V.S., Sibata C.H. (2010). Future of oncologic photodynamic therapy. Future Oncol..

[4] Yano S., Hirohara S., Obata M., Hagiya Y., Ogura S., Ikeda I., Kataoka H., Tanaka M., Joh T. (2011). Current states and future views in photodynamic therapy. J. Photochem. Photobiol. C.

[5] Dabrowski J.M., Arnaut L.G. (2015). Photodynamic therapy (PDT) of cancer: From local to systemic treatment. Photochem Photobiol Sci..

[6] Zhu J., Dominijanni A., Rodríguez-Corrales J.Á., Prussin R., Zhao Z., Li T., Robertson J.L., Brewer K.J. (2017). Visible light-induced cytotoxicity of Ru,Os–polyazine complexes towards rat malignant glioma. Inorg. Chim. Acta.

[7] Marian C.M. (2012). Spin-orbit coupling and intersystem crossing in molecules. Wiley Interdiscip. Rev. Comput. Mol. Sci..

[8] Banfi S., Caruso E., Caprioli S., Mazzagatti L., Canti G., Ravizza R., Gariboldi M., Monti E. (2004). Photodynamic effects of porphyrin and chlorin photosensitizers in human colon adenocarcinoma cells. Bioorg. Med. Chem..

[9] García-Díaz M., Sánchez-García D., Soriano J., Sagrista L., Mora M., Villanueva A., Stockert J., Cañete M., Nonell S. (2011). Temocene: The porphycene analogue of temoporfin (Foscan®). MedChemComm.

[10] Nanashima A., Abo T., Nonaka T., Nonaka Y., Morisaki T., Uehara R., Ohnita K., Fukuda D., Murakami G., Tou K. (2012). Photodynamic therapy using talaporfin sodium (Laserphyrin^®^) for bile duct carcinoma: A preliminary clinical trial. Anticancer. Res..

[11] O’Connor A.E., Gallagher W.M., Byrne A.T. (2009). Porphyrin and Nonporphyrin Photosensitizers in Oncology: Preclinical and Clinical Advances in Photodynamic Therapy. Photochem. Photobiol..

[12] Liu M., Chen C.-Y., Hood D., Taniguchi M., Diers J.R., Bocian D.F., Holten D., Lindsey J.S. (2017). Synthesis, photophysics and electronic structure of oxobacteriochlorins. New J. Chem..

[13] Tamiaki H., Xu M., Kinoshita Y. (2013). Synthesis of oxo-, thioxo- and methylene-substituted bacteriochlorins by modifying chlorophyll-a and their electronic absorption in visible and near-infrared regions. J. Photochem. Photobiol. A Chem..

[14] Kamkaew A., Lim S.H., Lee H.B., Kiew L.V., Chung L.Y., Burgess K. (2013). BODIPY dyes in photodynamic therapy. Chem. Soc. Rev..

[15] Ramaiah D., Eckert I., Arun K.T., Weidenfeller L., Epe B. (2002). Squaraine dyes for photodynamic therapy: Study of their cytotoxicity and genotoxicity in bacteria and mammalian cells. Photochem. Photobiol..

[16] Loudet A., Burgess K. (2007). BODIPY dyes and their derivatives: Syntheses and spectroscopic properties. Chem. Rev..

[17] Patalag L.J., Jones P.G., Werz D.B. (2016). BOIMPYs: Rapid Access to a Family of Red-Emissive Fluorophores and NIR Dyes. Angew. Chem., Int. Ed..

[18] Kasha M. (1950). Characterization of electronic transitions in complex molecules. Discuss Faraday Soc..

[19] El-Sayed M.A. (1968). Triplet state. Its radiative and nonradiative properties. Acc. Chem. Res..

[20] Pirillo J., Mazzone G., Russo N., Bertini L. (2017). Photophysical properties of S, Se and te-substituted deoxyguanosines: Insight into their ability to act as chemotherapeutic agents. J. Chem. Inf. Model.

[21] Pirillo J., De Simone B.C., Russo N. (2016). Photophysical properties prediction of seleniumand tellurium-substituted thymidine as potential UVA chemotherapeutic agents. Theor. Chem. Acc..

[22] De Simone B.C., Mazzone G., Pirillo J., Russo N., Sicilia E. (2017). Halogen atom effect on the photophysical properties of substituted aza-BODIPY derivatives. Phys. Chem. Chem. Phys..

[23] De Simone B.C., Mazzone G., Russo N., Sicilia E., Toscano M. (2018). Excitation energies, singlet–triplet energy gaps, spin–orbit matrix elements and heavy atom effects in BOIMPYs as possible photosensitizers for photodynamic therapy: A computational investigation. Phys. Chem. Chem. Phys..

[24] De Simone B.C., Mazzone G., Russo N., Sicilia E., Toscano M. (2017). Metal atom effect on the photophysical properties of Mg(II), Zn(II), Cd(II), and Pd(II) tetraphenylporphyrin complexes proposed as possible drugs in photodynamic therapy. Molecules.

[25] Frisch M.J., Trucks G.W., Schlegel H.B., Scuseria G.E., Robb M.A., Cheeseman J.R., Scalmani G., Barone V., Mennucci B., Petersson G.A. (2010). Gaussian 09, Revision D.01.

[26] Casida M.E., Chong D.P. (1995). Recent Advances in Density Functional Methods, Part I.

[27] Becke A.D. (1993). Density-functional thermochemistry. III. The role of exact exchange. J. Chem. Phys..

[28] Lee C., Yang W., Parr R.G. (1988). Development of the colle-salvetti correlation-energy formula into a functional of the electron density. Phys. Rev. B Condens. Matter Mater. Phys..

[29] Andrae D., Haussermann U., Dolg M., Stoll H., Preuss H. (1990). Energy-adjusted ab initio pseudopotentials for the second and third row transition elements. Theor. Chim. Acta.

[30] Cossi M., Barone V. (2000). Solvent effect on vertical electronic transitions by the polarizable continuum model. J. Chem. Phys..

[31] Dalton (2011). A Molecular Electronic Structure Program. Release Dalton. http://daltonprogram.org/.

[32] Ågren H., Vahtras O., Minaev B. (1996). Response theory and calculations of spin-orbit coupling phenomena in molecules. Adv. Quantum Chem..

[33] Koseki S., Schmidt M.W., Gordon M.S. (1998). Effective nuclear charges for the first- through third-row transition metal elements in spin–orbit calculations. J. Phys. Chem. A.

[34] De Simone B.C., Mazzone G., Russo N., Sicilia E., Toscano M. (2018). Computational investigation of the influence of halogen atoms on the photophysical properties of tetraphenylporphyrin and its Zinc(II) complexes. J. Phys. Chem. A.

[35] Alberto M.E., De Simone B.C., Mazzone G., Marino T., Russo N. (2015). Photophysical properties of free and metallated mesosubstituted tetrabenzotriazaporphyrin from density functional theory investigation. Dyes Pigm..

[36] De Simone B.C., Mazzone G., Sang-aroon W., Marino T., Russo N., Sicilia E. (2019). Theoretical insight into joint photodynamic action of a gold(I) complex and a BODIPY chromophore for singlet oxygen generation. Phys. Chem. Chem. Phys..

[37] Alberto M.E., De Simone B.C., Mazzone G., Sicilia E., Russo N. (2015). The heavy atom effect on Zn(II) phthalocyanine derivatives: A theoretical exploration of the photophysical properties. Phys. Chem. Chem. Phys..

[38] Alberto M.E., Iuga C., Quartarolo A.D., Russo N. (2013). Bisanthracene Bis(dicarboxylic imide)s as potential photosensitizers in Photodynamic therapy. A theoretical investigation. J. Chem. Inf. Model..

[39] Alberto M.E., De Simone B.C., Russo N., Sicilia E., Toscano M. (2018). Can BODIPY Dimers Act as Photosensitizers in Photodynamic Therapy? A Theoretical Prediction. Front. Phys..

