# Supplementary material for: Rational Design of Modified Oxobacteriochlorins as Potential Photodynamic Therapy Photosensitizers
Source: Int J Mol Sci. 2019 Apr 24;20(8):2002. doi: 10.3390/ijms20082002 (PMC6514987; doi:10.3390/ijms20082002)
Supplement: Supplementary file 1 [file ijms-20-02002-s001.pdf]

Supporting Information

Rational design of modified oxobacteriochlorins as potential photodynamic therapy photosensitizers

Marta Erminia Alberto, Bruna Clara De Simone, Emilia Sicilia, Marirosa Toscano and Nino Russo*

Dipartimento di Chimica e Tecnologie Chimiche, Università della Calabria, 87036 Rende, Italy

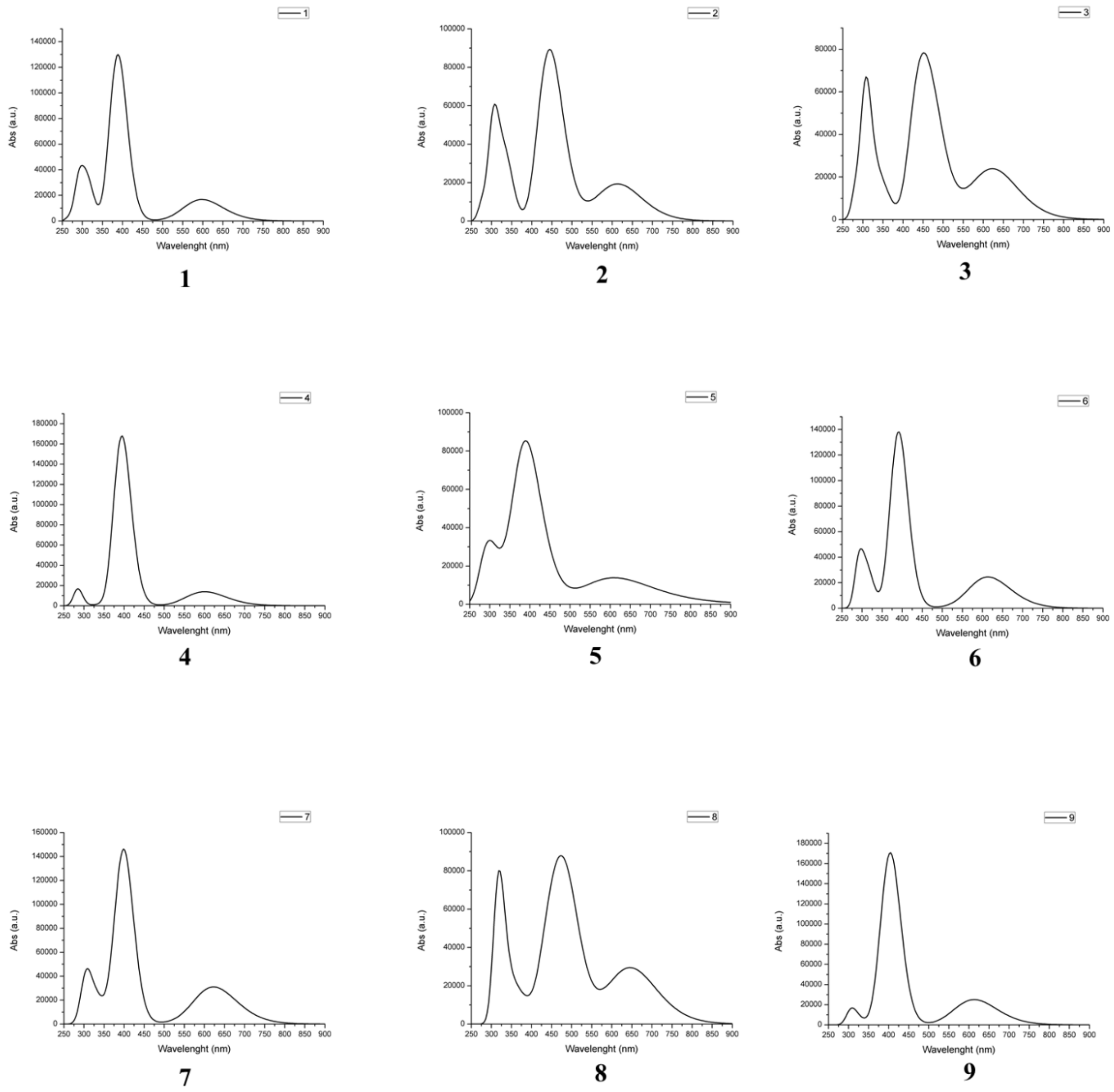

Figure S1. Simulated electronic absorption spectra

-Table S1: Cartesian Coordinates of Optimized structures................................

1

	X	Y	Z
C	1.16475300	2.73141700	0.07261400
C	0.81301800	4.11392800	0.00860200
C	-0.55656400	4.21190000	0.04125800
C	-1.09786300	2.89287700	0.12491500
N	-0.02215500	2.03263300	0.15038000
H	-0.08923700	1.02394100	0.17533900
H	1.52100400	4.92570300	-0.06025300
H	-1.14311600	5.11726500	-0.00083800
C	-2.46831200	2.55016000	0.13523500
C	-3.01443900	1.26235000	0.15413000
C	-4.53096600	1.00278300	0.12432800
N	-2.31865300	0.10194300	0.09776300
C	-4.60461300	-0.49905500	0.44061700
C	-3.18043700	-0.94441700	0.13127600
C	-2.85544600	-2.29240100	-0.05723500
C	-1.55415800	-2.82106400	-0.20101500
C	-1.20150700	-4.20050500	-0.32612800
N	-0.36897500	-2.12008100	-0.15227000
C	0.16812000	-4.29522900	-0.32933500
H	-1.91058800	-5.01246600	-0.38048800
C	0.70828000	-2.97842200	-0.20347500
H	-0.30566600	-1.11645300	-0.04552400
H	0.75916100	-5.19662500	-0.39172400
C	2.07141000	-2.64389300	-0.07671000
C	2.61135500	-1.36176600	0.06687800
C	4.09585500	-1.15372000	0.34950700
N	1.93356700	-0.19028200	0.03406000
C	4.25706800	0.34175000	-0.01762700
C	2.80985100	0.84147200	0.05613800
C	2.47170100	2.19842100	0.03454400
H	3.27936900	2.89846700	-0.01553500
H	-3.16368200	3.36336700	0.12779700
H	-4.84137400	-0.66207300	1.47128200
H	-3.66851800	-2.98688600	-0.09601200
H	2.76690200	-3.45688700	-0.09193000
C	4.96262900	-2.04366200	-0.56060600
H	5.97117900	-1.68635600	-0.55298400
H	4.93853000	-3.05138100	-0.20168700
H	4.58041700	-2.01158700	-1.55949800
C	4.41555800	-1.39625200	1.83630500
H	4.42772400	-2.44822400	2.03151600
H	5.37347600	-0.97944600	2.06774600
H	3.66760300	-0.92959900	2.44268700
O	5.44599300	1.06748500	-0.34114800
H	-5.30709000	-0.99104200	-0.19920700

C	-5.25019700	1.82760100	1.20783300
H	-5.31274700	2.84912300	0.89561400
H	-6.23603000	1.43899000	1.35620100
H	-4.70153700	1.76777800	2.12450900
C	-5.11094800	1.29867200	-1.27126200
H	-6.08814700	0.86990800	-1.34963300
H	-5.17337600	2.35720700	-1.41446300
H	-4.47429400	0.87448600	-2.01935200

2

	X	Y	Z
C	1.14457200	2.72097900	0.12455900
C	0.79516600	4.10871700	0.09113400
C	-0.57772500	4.20404100	0.04454000
C	-1.11629000	2.87641300	0.04828800
N	-0.04828400	2.02040800	0.09773800
H	-0.12070600	1.01103800	0.11040400
H	1.51449500	4.91750600	0.10157200
H	-1.17670100	5.10501700	0.01022500
C	-2.47636400	2.51875100	0.00724700
C	-3.04240000	1.25171000	0.01223400
C	-4.56629200	1.03526200	-0.05194400
N	-2.33859900	0.09093800	0.06041700
C	-4.64909500	-0.51057900	0.02853400
C	-3.19542700	-0.94948300	0.06010300
C	-2.83047900	-2.29793200	0.09128700
C	-1.54610300	-2.84773500	0.13097900
C	-1.20207700	-4.24262300	0.16108200
N	-0.36018900	-2.15454600	0.15031700
C	0.16697300	-4.34482500	0.19764100
H	-1.92534400	-5.04787600	0.15507300
C	0.71580800	-3.01715500	0.19193500
H	-0.28583200	-1.14529200	0.13782900
H	0.76158800	-5.24900100	0.22687700
C	2.06056400	-2.66240600	0.22426300
C	2.62217000	-1.37465600	0.22348400
C	4.13181200	-1.14088200	0.26579500
N	1.92336000	-0.23571500	0.18795300
C	4.18377100	0.38471500	0.24925500
C	2.79842800	0.82838600	0.20115400
C	2.42413600	2.17631300	0.17198000
H	3.24427300	2.88850100	0.18707600
H	-3.15996600	3.36186300	-0.03479800
H	-5.17531900	-0.84947600	0.93030400
H	-3.64211900	-3.02091100	0.08479000
H	2.75308700	-3.49864500	0.25608800
C	4.84337400	-1.72864100	-0.97461400
H	5.89786600	-1.43345700	-0.96204900
H	4.78517800	-2.82278000	-0.97094300

H	4.39266600	-1.36200100	-1.90303000
C	4.76712400	-1.69905500	1.56008300
H	4.71095600	-2.79313500	1.57712000
H	5.81997600	-1.40144200	1.60549400
H	4.25994100	-1.31328800	2.45089400
H	-5.17741200	-0.94666500	-0.82827000
C	-5.28814100	1.70716800	1.13292800
H	-5.18330900	2.79787800	1.10154200
H	-6.36006800	1.47381400	1.10495600
H	-4.88776200	1.35751700	2.09156000
C	-5.13864000	1.56052600	-1.38540400
H	-6.21090000	1.33685100	-1.45227400
H	-5.01573700	2.64626700	-1.47310500
H	-4.63858800	1.09458400	-2.24251300
S	5.56281100	1.30131800	0.28099600

3

	X	Y	Z
C	1.14601400	2.72061600	0.12402500
C	0.79561700	4.10896200	0.09069500
C	-0.57663200	4.20321500	0.04480700
C	-1.11485600	2.87462500	0.04859900
N	-0.04725900	2.01936000	0.09756000
H	-0.11850900	1.00990500	0.11016400
H	1.51482600	4.91784500	0.10085800
H	-1.17652700	5.10358900	0.01081700
C	-2.47510400	2.51781900	0.00778600
C	-3.04105500	1.25077200	0.01240200
C	-4.56474300	1.03403500	-0.05165500
N	-2.33675800	0.09008800	0.06020900
C	-4.64712900	-0.51179900	0.02751700
C	-3.19349600	-0.95030800	0.05936700
C	-2.82873500	-2.29895800	0.09025700
C	-1.54425900	-2.84795500	0.13021200
C	-1.19925400	-4.24309600	0.16023700
N	-0.35901700	-2.15402800	0.14995300
C	0.16938700	-4.34446700	0.19739300
H	-1.92218900	-5.04864000	0.15381700
C	0.71762000	-3.01608000	0.19197700
H	-0.28514600	-1.14471100	0.13758500
H	0.76467500	-5.24818100	0.22679600
C	2.06094400	-2.66040000	0.22471700
C	2.62047000	-1.37082200	0.22370600
C	4.12996400	-1.13554400	0.26591000
N	1.92170200	-0.23374000	0.18796500
C	4.17643200	0.38508300	0.24749300
C	2.80188600	0.82928600	0.20049100
C	2.42427100	2.17839600	0.17094200
H	3.24386600	2.89133200	0.18547000
H	-3.15844800	3.36110900	-0.03386700

H	-5.17362000	-0.85177300	0.92871400
H	-3.64030200	-3.02196100	0.08337600
H	2.75482300	-3.49532800	0.25692100
C	4.83936100	-1.72794100	-0.97397700
H	5.90020200	-1.45721000	-0.95142000
H	4.75657700	-2.82056200	-0.98108800
H	4.40375300	-1.34091800	-1.90095600
C	4.76253600	-1.69547300	1.56133100
H	4.67789800	-2.78753900	1.59155700
H	5.82321200	-1.42560500	1.59613000
H	4.27219500	-1.28478800	2.45002900
H	-5.17477200	-0.94747100	-0.82990800
C	-5.28630200	1.70485700	1.13404300
H	-5.18167500	2.79558800	1.10342400
H	-6.35815600	1.47126100	1.10612800
H	-4.88559200	1.35450800	2.09227500
C	-5.13748000	1.56042400	-1.38452900
H	-6.20962800	1.33630600	-1.45133700
H	-5.01509700	2.64629300	-1.47107900
H	-4.63741100	1.09556900	-2.24220900
Se	5.67083000	1.37095900	0.27948700

4

	X	Y	Z
C	1.16475300	2.73141700	0.07261400
C	0.81301800	4.11392800	0.00860200
C	-0.55656400	4.21190000	0.04125800
C	-1.09786300	2.89287700	0.12491500
N	-0.02215500	2.03263300	0.15038000
H	-0.08923700	1.02394100	0.17533900
H	1.52100400	4.92570300	-0.06025300
C	-2.46831200	2.55016000	0.13523500
C	-3.01443900	1.26235000	0.15413000
C	-4.53096600	1.00278300	0.12432800
N	-2.31865300	0.10194300	0.09776300
C	-4.60461300	-0.49905500	0.44061700
C	-3.18043700	-0.94441700	0.13127600
C	-2.85544600	-2.29240100	-0.05723500
C	-1.55415800	-2.82106400	-0.20101500
C	-1.20150700	-4.20050500	-0.32612800
N	-0.36897500	-2.12008100	-0.15227000
C	0.16812000	-4.29522900	-0.32933500
C	0.70828000	-2.97842200	-0.20347500
H	-0.30566600	-1.11645300	-0.04552400
H	0.75916100	-5.19662500	-0.39172400
C	2.07141000	-2.64389300	-0.07671000
C	2.61135500	-1.36176600	0.06687800
C	4.09585500	-1.15372000	0.34950700
N	1.93356700	-0.19028200	0.03406000

C	4.25706800	0.34175000	-0.01762700
C	2.80985100	0.84147200	0.05613800
C	2.47170100	2.19842100	0.03454400
H	3.27936900	2.89846700	-0.01553500
H	-3.16368200	3.36336700	0.12779700
H	-3.66851800	-2.98688600	-0.09601200
H	2.76690200	-3.45688700	-0.09193000
C	4.96262900	-2.04366200	-0.56060600
H	5.97117900	-1.68635600	-0.55298400
H	4.93853000	-3.05138100	-0.20168700
H	4.58041700	-2.01158700	-1.55949800
C	4.41555800	-1.39625200	1.83630500
H	4.42772400	-2.44822400	2.03151600
H	5.37347600	-0.97944600	2.06774600
H	3.66760300	-0.92959900	2.44268700
O	5.44599300	1.06748500	-0.34114800
O	-5.71467600	-1.27319000	0.90254200
C	-2.21319800	-5.35898100	-0.40368700
H	-2.77802200	-5.40062900	0.50413400
H	-2.87548700	-5.19949400	-1.22881500
H	-1.68901100	-6.28173500	-0.54026800
C	-1.39326600	5.50338100	-0.01879100
H	-1.98513000	5.50378800	-0.91019300
H	-2.03539100	5.55240300	0.83571000
H	-0.74062200	6.35128000	-0.02361300
C	-5.25019700	1.82760100	1.20783300
H	-5.23313400	2.86287600	0.93799100
H	-6.26456000	1.49776200	1.29252200
H	-4.75262000	1.69525200	2.14581000
C	-5.11094800	1.29867200	-1.27126200
H	-6.08959300	0.87298900	-1.34837900
H	-5.17002600	2.35721000	-1.41585400
H	-4.47619800	0.87140200	-2.01921400

5

	X	Y	Z
C	1.16475300	2.73141700	0.07261400
C	0.81301800	4.11392800	0.00860200
C	-0.55656400	4.21190000	0.04125800
C	-1.09786300	2.89287700	0.12491500
N	-0.02215500	2.03263300	0.15038000
H	-0.08923700	1.02394100	0.17533900
H	-1.14311600	5.11726500	-0.00083800
C	-2.46831200	2.55016000	0.13523500
C	-3.01443900	1.26235000	0.15413000
C	-4.53096600	1.00278300	0.12432800
N	-2.31865300	0.10194300	0.09776300
C	-4.60461300	-0.49905500	0.44061700
C	-3.18043700	-0.94441700	0.13127600
C	-2.85544600	-2.29240100	-0.05723500

C	-1.55415800	-2.82106400	-0.20101500
C	-1.20150700	-4.20050500	-0.32612800
N	-0.36897500	-2.12008100	-0.15227000
C	0.16812000	-4.29522900	-0.32933500
C	0.70828000	-2.97842200	-0.20347500
H	-0.30566600	-1.11645300	-0.04552400
H	0.75916100	-5.19662500	-0.39172400
C	2.07141000	-2.64389300	-0.07671000
C	2.61135500	-1.36176600	0.06687800
C	4.09585500	-1.15372000	0.34950700
N	1.93356700	-0.19028200	0.03406000
C	4.25706800	0.34175000	-0.01762700
C	2.80985100	0.84147200	0.05613800
C	2.47170100	2.19842100	0.03454400
H	3.27936900	2.89846700	-0.01553500
H	-3.16368200	3.36336700	0.12779700
H	-4.84137400	-0.66207300	1.47128200
H	-3.66851800	-2.98688600	-0.09601200
H	2.76690200	-3.45688700	-0.09193000
C	4.96262900	-2.04366200	-0.56060600
H	5.97117900	-1.68635600	-0.55298400
H	4.93853000	-3.05138100	-0.20168700
H	4.58041700	-2.01158700	-1.55949800
C	4.41555800	-1.39625200	1.83630500
H	4.42772400	-2.44822400	2.03151600
H	5.37347600	-0.97944600	2.06774600
H	3.66760300	-0.92959900	2.44268700
O	5.44599300	1.06748500	-0.34114800
H	-5.30709000	-0.99104200	-0.19920700
Br	2.06587600	5.55045200	-0.11324400
Br	-2.45626600	-5.63731600	-0.42232100
C	-5.25019700	1.82760100	1.20783300
H	-5.31948200	2.84765600	0.89225900
H	-6.23332500	1.43426200	1.36159600
H	-4.69750700	1.77397200	2.12246900
C	-5.11094800	1.29867200	-1.27126200
H	-6.06659000	0.82712700	-1.36766200
H	-5.22072900	2.35579800	-1.39508300
H	-4.44849800	0.91867500	-2.02070300

6

	X	Y	Z
C	1.15486800	2.73833200	0.11976400
C	0.78388600	4.12130700	0.08307200
C	-0.59003700	4.21629700	0.03407300
C	-1.11252300	2.88642200	0.03960500
N	-0.03739300	2.03813200	0.09184300
H	-0.10145700	1.02727100	0.10671900
H	-1.18994700	5.11520900	-0.00276700

C	-2.47100700	2.52277400	-0.00190800
C	-3.03599900	1.25517100	0.00307300
C	-4.56050500	1.04189200	-0.05657500
N	-2.33279900	0.09381200	0.05135800
C	-4.64491000	-0.50446800	0.00515300
C	-3.19176900	-0.94469800	0.04729700
C	-2.83730300	-2.29604300	0.08147100
C	-1.56107600	-2.85877900	0.12758100
C	-1.19792800	-4.25149600	0.16290300
N	-0.36909200	-2.17099700	0.14953900
C	0.17102900	-4.35805600	0.20402300
C	0.70704300	-3.02960700	0.19605400
H	-0.29808100	-1.16071100	0.13422800
H	0.76449500	-5.26151700	0.23751100
C	2.05674400	-2.67590700	0.22986900
C	2.61941600	-1.39504500	0.22659900
C	4.12913900	-1.16583200	0.26803500
N	1.90869700	-0.25169300	0.18847000
C	4.17306000	0.36540100	0.24976700
C	2.75968300	0.81727000	0.19986600
C	2.42688300	2.16725000	0.17028600
H	3.26821900	2.85407300	0.18770600
H	-3.15673400	3.36407300	-0.04423700
H	-5.18290400	-0.85484900	0.89523900
H	-3.65697700	-3.00802700	0.07135300
H	2.74684900	-3.51425900	0.26446800
C	4.85414000	-1.72458100	-0.97506200
H	5.90081900	-1.40100100	-0.96540800
H	4.83020100	-2.82007400	-0.97783100
H	4.39217400	-1.37012400	-1.90316800
C	4.78047000	-1.69351000	1.56418500
H	4.76224900	-2.78881900	1.58889800
H	5.82413800	-1.36353500	1.61079500
H	4.26138000	-1.32261300	2.45495900
O	5.17919600	1.06208400	0.27135700
H	-5.16146900	-0.92980900	-0.86414500
C	-5.27192400	1.69844500	1.14380000
H	-5.16382800	2.78926400	1.12807900
H	-6.34468400	1.46858100	1.11967700
H	-4.86607000	1.33296500	2.09426800
C	-5.14247600	1.58581100	-1.37823800
H	-6.21505300	1.36243900	-1.44000200
H	-5.02138000	2.67285000	-1.45204000
H	-4.64893800	1.13197600	-2.24561000
I	2.12807700	5.74894300	0.09945200
I	-2.55654600	-5.86846900	0.15378500

7

	X	Y	Z
C	1.06364700	2.76739900	0.17993600
C	0.70643000	4.14469400	0.20879600
C	-0.67494800	4.25273600	0.15048200
C	-1.19681400	2.90923800	0.06278300
N	-0.11944600	2.06158400	0.09593800
H	-0.19636500	1.05324600	0.03809000
H	1.41537700	4.95793200	0.29969100
C	-2.53672100	2.51139300	-0.08772900
C	-3.05403400	1.22495000	-0.17456100
C	-4.55759300	0.96127800	-0.38446400
N	-2.31743200	0.08503100	-0.12084500
C	-4.60658000	-0.57912000	-0.23101500
C	-3.14172600	-0.98063900	-0.18780200
C	-2.74615900	-2.31903600	-0.19819600
C	-1.45519200	-2.85756800	-0.16117200
C	-1.10602500	-4.24332100	-0.19625900
N	-0.27228900	-2.16191200	-0.10176300
C	0.27107000	-4.36203800	-0.16771400
H	-1.82316200	-5.05075000	-0.27350500
C	0.80754100	-3.01880200	-0.08794300
H	-0.18892700	-1.15419000	-0.04412000
C	2.14004900	-2.63110800	0.03965600
C	2.65632900	-1.33208600	0.13767900
C	4.15286300	-1.05804100	0.27788100
N	1.91285200	-0.21118600	0.11798300
C	4.14849200	0.47384400	0.31352800
C	2.72715100	0.88358700	0.21973900
C	2.34882100	2.22106600	0.23957100
H	3.16668200	2.93346200	0.31288100
H	-3.24834100	3.32731100	-0.15342600
H	-5.10710000	-0.87820900	0.70004700
H	-3.54459200	-3.05535000	-0.24560500
H	2.85804400	-3.44348100	0.07839200
C	4.97382100	-1.55523300	-0.92966800
H	6.00553200	-1.19623300	-0.84508300
H	4.99046200	-2.65043600	-0.96317700
H	4.55846800	-1.18987600	-1.87548200
C	4.73568800	-1.60770100	1.59775900
H	4.74560100	-2.70336800	1.58983000
H	5.76462900	-1.25190700	1.72160900
H	4.15006000	-1.27766900	2.46297700
O	5.13304400	1.19771600	0.40390900
H	-5.14226400	-1.07039700	-1.05185900
C	1.04188700	-5.61831300	-0.20490500
C	2.14105000	-5.79100900	-1.06495400
C	0.66268400	-6.70762600	0.60097700
C	2.83524400	-7.00162300	-1.10719900
H	2.43414300	-4.98437200	-1.73140000
C	1.35964800	-7.91439700	0.55259700
H	-0.17886900	-6.59898500	1.28056400

C	2.46362300	-8.08447900	-0.29753500
H	3.67463600	-7.10834200	-1.79135800
H	1.04328800	-8.73709000	1.19092800
C	-1.45352900	5.50544900	0.16896900
C	-1.06531300	6.59452000	-0.63288800
C	-2.56902100	5.67646400	1.00809900
C	-1.76799000	7.79881600	-0.60022400
H	-0.21018500	6.48856000	-1.29582700
C	-3.27001600	6.88372500	1.03370800
H	-2.87057500	4.87035500	1.67149500
C	-2.88826000	7.96639700	0.22848200
H	-1.44253200	8.62158900	-1.23393100
H	-4.12236600	6.98844300	1.70217800
C	3.23588500	-9.38333200	-0.32155100
H	3.78269400	-9.50649200	-1.26251300
H	3.97205500	-9.42012500	0.49304900
H	2.57244300	-10.24675200	-0.19779900
C	-3.66663400	9.26201100	0.23531800
H	-4.40067900	9.28689800	-0.58175700
H	-4.21698500	9.39329600	1.17324600
H	-3.00666700	10.12699900	0.10435600
C	-5.44017300	1.67310500	0.65674700
H	-5.38310900	2.76328500	0.55879700
H	-6.49036600	1.38408000	0.52303000
H	-5.14189800	1.40840700	1.67783700
C	-4.97191400	1.38997900	-1.80960800
H	-6.02633700	1.14397800	-1.98937100
H	-4.84652300	2.46974900	-1.95108100
H	-4.36765700	0.87841000	-2.56800800

8

	X	Y	Z
C	1.07277600	2.75627600	0.19372300
C	0.71136400	4.13674500	0.21997700
C	-0.66604400	4.24314300	0.15471500
C	-1.18728200	2.89591900	0.06453200
N	-0.11253600	2.04952700	0.10347200
H	-0.18468400	1.04104200	0.04677700
H	1.42022400	4.94978100	0.31305500
C	-2.52889700	2.50520400	-0.08746100
C	-3.05112300	1.22101300	-0.17283500
C	-4.55566900	0.96392800	-0.37996300
N	-2.31873200	0.07823600	-0.11827800
C	-4.61079300	-0.57631000	-0.22936000
C	-3.14839300	-0.98332700	-0.18458500
C	-2.75674900	-2.32316300	-0.19494600
C	-1.46425000	-2.85614100	-0.15868800
C	-1.10858800	-4.24203200	-0.19453200
N	-0.28620300	-2.15453200	-0.09941400
C	0.26713100	-4.35461100	-0.16691900

H	-1.82274600	-5.05197100	-0.27229200
C	0.79883400	-3.00646700	-0.08529100
H	-0.21216300	-1.14624100	-0.04143200
C	2.12556300	-2.61740900	0.04346900
C	2.64218500	-1.31266700	0.14513200
C	4.13994400	-1.04209300	0.28308100
N	1.91467000	-0.19357900	0.13107100
C	4.14577000	0.47741900	0.33697100
C	2.76409900	0.88953000	0.24163900
C	2.35703600	2.22976400	0.25912200
H	3.16163700	2.95576900	0.33551200
H	-3.23628700	3.32448700	-0.15398800
H	-5.11360300	-0.87579000	0.70026800
H	-3.55485600	-3.05949800	-0.24208600
H	2.84521100	-3.42777100	0.08125400
C	4.93367700	-1.55774600	-0.93933200
H	5.98323400	-1.26022400	-0.84438200
H	4.88344700	-2.65061000	-1.00022900
H	4.53954200	-1.13844500	-1.87091300
C	4.71293300	-1.64585400	1.58671500
H	4.65808300	-2.73993300	1.56275900
H	5.76140200	-1.34922800	1.69612700
H	4.16007600	-1.28973600	2.46218500
H	-5.14629400	-1.06491000	-1.05184900
C	1.04451300	-5.60619900	-0.20514000
C	2.14883400	-5.76969200	-1.06044700
C	0.66554300	-6.70067200	0.59392100
C	2.84837600	-6.97699800	-1.10448900
H	2.44115900	-4.95949600	-1.72278800
C	1.36853100	-7.90369300	0.54412800
H	-0.17999200	-6.59893600	1.26957600
C	2.47763400	-8.06487900	-0.30115800
H	3.69160800	-7.07696200	-1.78484000
H	1.05290400	-8.73053400	1.17735800
C	-1.44798600	5.49419600	0.16612000
C	-1.06563000	6.57683300	-0.64677800
C	-2.55923000	5.66926800	1.00972100
C	-1.77048600	7.78003900	-0.62017200
H	-0.21357000	6.46691700	-1.31299300
C	-3.26199500	6.87556100	1.02959200
H	-2.85625500	4.86760700	1.68058200
C	-2.88637900	7.95221600	0.21340300
H	-1.44999800	8.59823000	-1.26221400
H	-4.11091100	6.98429600	1.70171700
C	3.25618600	-9.35979200	-0.32644500
H	3.80701800	-9.47737500	-1.26572800
H	3.98952600	-9.39508200	0.49073200
H	2.59649900	-10.22671500	-0.20748300
C	-3.66717100	9.24634400	0.21381600
H	-4.40557300	9.26290000	-0.59950900
H	-4.21270700	9.38433200	1.15356200

H	-3.00957400	10.11139300	0.07205400
C	-5.43179300	1.67789800	0.66540700
H	-5.36990100	2.76786900	0.56915300
H	-6.48349600	1.39372300	0.53396700
H	-5.13187500	1.40991100	1.68512800
C	-4.97149100	1.39766800	-1.80326900
H	-6.02725500	1.15612200	-1.98052000
H	-4.84239800	2.47720900	-1.94239200
H	-4.37129600	0.88554000	-2.56447300
Se	5.61146400	1.49649300	0.48989700

9

	X	Y	Z
C	1.05555800	2.76730700	0.15905100
C	0.70488600	4.15027400	0.19017500
C	-0.67199500	4.26614400	0.12201000
C	-1.20141900	2.92208200	0.02273700
N	-0.12654900	2.06803600	0.06082600
H	-0.20973000	1.06012900	-0.00004100
H	1.41827900	4.95824900	0.29089400
C	-2.53514000	2.53135900	-0.13613200
C	-3.04753600	1.23709800	-0.24122100
C	-4.54079400	0.96230400	-0.41187700
N	-2.30152300	0.11120700	-0.20164900
C	-4.53541400	-0.56814900	-0.43559300
C	-3.11074300	-0.97889300	-0.31284400
C	-2.74009200	-2.32037400	-0.32279700
C	-1.45872100	-2.87057200	-0.24648700
C	-1.10805200	-4.25354000	-0.27761700
N	-0.27661900	-2.17130800	-0.14820700
C	0.26882900	-4.36941400	-0.20942300
C	0.79825100	-3.02535900	-0.11011600
H	-0.19342800	-1.16340000	-0.08735700
C	2.13197000	-2.63463200	0.04877500
C	2.64436300	-1.34037000	0.15384900
C	4.13761600	-1.06557500	0.32454000
N	1.89835300	-0.21447700	0.11425000
C	4.13225200	0.46488000	0.34810100
C	2.70757500	0.87562300	0.22539700
C	2.33692900	2.21710600	0.23532800
H	3.15758900	2.92468300	0.31955000
H	-3.25141200	3.34417500	-0.19165900
H	-3.56074600	-3.02795200	-0.40706300
H	2.84824000	-3.44744700	0.10432000
C	4.98617900	-1.57328100	-0.85963200
H	6.01587600	-1.21471700	-0.75340700
H	5.00235800	-2.66858100	-0.88265100
H	4.59379800	-1.21604800	-1.81817600
C	4.69019900	-1.60406300	1.66255600
H	4.69927400	-2.69959100	1.66310300
H	5.71635500	-1.24797200	1.80597800

H	4.08562800	-1.26694900	2.51172500
O	5.10942400	1.19339700	0.45063100
O	-5.51257400	-1.29666300	-0.53825700
H	-1.82144100	-5.06151400	-0.37836500
C	1.04412900	-5.62358500	-0.22933300
C	2.16186100	-5.79264500	-1.06569800
C	0.65008300	-6.71346700	0.56826700
C	2.85998800	-7.00130700	-1.09316800
H	2.46629200	-4.98561800	-1.72667600
C	1.35135300	-7.91826400	0.53469500
H	-0.20684800	-6.60765500	1.22879300
C	2.47368000	-8.08515000	-0.29160600
H	3.71364400	-7.10608700	-1.75969700
H	1.02312300	-8.74215600	1.16528300
C	-1.44730300	5.52030800	0.14192100
C	-1.05327600	6.61019500	-0.65567900
C	-2.56502900	5.68935600	0.97829900
C	-1.75455600	7.81498700	-0.62209500
H	-0.19635100	6.50439800	-1.31621500
C	-3.26316300	6.89801300	1.00578200
H	-2.86944600	4.88232300	1.63927600
C	-2.87687100	7.98186400	0.20422200
H	-1.42633900	8.63887700	-1.25269200
H	-4.11681400	7.00278700	1.67231800
C	3.24936300	-9.38207600	-0.29997300
H	3.80418400	-9.51059500	-1.23550700
H	3.97898900	-9.41083800	0.52081100
H	2.58703400	-10.24608400	-0.17532100
C	-3.65254300	9.27879700	0.21263200
H	-4.38190700	9.30775900	-0.60837500
H	-4.20765000	9.40711300	1.14802500
H	-2.99015900	10.14282300	0.08838600
C	-5.09352100	1.50098400	-1.74973600
H	-6.11968200	1.14489000	-1.89311200
H	-5.10262900	2.59651200	-1.75011900
H	-4.48903000	1.16401700	-2.59902200
C	-5.38924300	1.46981400	0.77248000
H	-4.99676500	1.11240900	1.73092000
H	-5.40540700	2.56510900	0.79569200
H	-6.41895300	1.11127300	0.66629500
